# Empirical Modeling of the Effect of Water Content on the Tensile Modulus of Polyamide‑6

**DOI:** 10.1021/acsomega.6c01154

**Published:** 2026-05-20

**Authors:** Ali Zarbali, Alina Tilekkabylova, Alfréd Menyhárd

**Affiliations:** Laboratory of Plastics and Rubber Technology, 61810 Department of Physical Chemistry and Materials Science at Budapest University of Technology and Economics, Műegyetem rkp. 3. H/1, Budapest 1111, Hungary

## Abstract

This work aims to model the effect of water content on the stiffness of polyamide-6. Our empirical model developed earlier was used to model stiffness. The tensile modulus is calculated from parameters of the crystalline structure obtained by calorimetry. The model was fitted to modulus data estimated after conditioning standard specimens in desiccators at different but constant relative humidities to change the water content over the widest range. The mechanical properties were studied by standard tensile tests, and the parameters of the crystalline structure were studied by calorimetry. The calculated modulus values are in good agreement with the experimentally measured data, indicating that the prediction of the modulus is accurate. The effect of water content was also described accurately, and one of the iterative parameters of our model showed a strong correlation with the water content of the sample. We assumed that this parameter weighs the intermolecular interactions of the polymer. According to the results of this study, the stiffness of any polyamide-6 grade can be calculated at any water content if experimental data are recorded at a single water content, which makes it possible to model the tensile modulus of a polyamide-6 specimen under realistic outdoor conditions.

## Graphical abstract

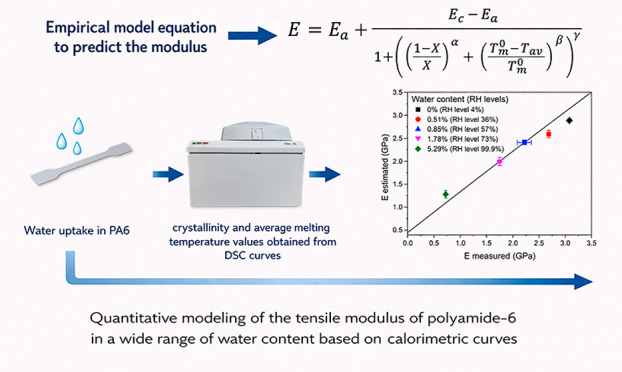

## Introduction

Polyamides are a class of polymeric materials in which an amide bond is formed and connects the repeating units. These materials belong to the class of engineering polymers because of their advantageous properties.^1^ One of the most popular engineering thermoplastics is polyamide-6, which has excellent tensile strength, stiffness, good impact resistance, as well as strong fatigue resistance and chemical stability.^2^ It is made by ring-opening polymerization of caprolactam in the melt in the presence of an acid catalyst and water vapor.^3^ Polyamide-6 has a melting point of 223 °C and a relatively high glass transition temperature around 50–75 °C under dry conditions.^1,4^ Its beneficial properties make it an excellent material for automotive and textile applications as well.^4^ Polyamide-6 is a semicrystalline polymer,^5^ which has three different crystalline modifications (α-, β-, and γ-forms),^6^ but the commercially applied polyamide-6 crystallizes mostly in the α-modification. The mechanical properties of polyamide-6 depend on the crystalline structure, similarly to any other crystalline polymer. Although polyamide-6 is used mainly in textile and automotive applications, its moisture sensitivity limits its performance. It is known that polyamide-6 is a hygroscopic polymer^7^ and absorbs a considerable amount of water. The mechanical performance of polyamides is determined by the intermolecular hydrogen bonds between the amide groups. These interactions are affected by water uptake, since the water can dissociate the hydrogen bonding within polyamide-6.^8,9^ In addition, the hygroscopic behavior is accompanied by a remarkable change in the glass transition temperature. Consequently, almost all mechanical properties, including the tensile stiffness, also depend on the water content of the polymer.^7^ Accordingly, it is an interesting and important challenge to quantitatively model and predict the change in mechanical performance as a function of water content, since most of the outdoor application fields are affected by the changing humidity in the environment.

The prediction of mechanical properties is possible but challenging in practice. Only a few models exist, and these models differ in how they describe the material. The earliest approaches treat these materials as composites consisting of crystalline and amorphous phases.^10–13^ The crystalline phase has a higher density and reinforces the amorphous matrix. Other models handle the multiphasic semicrystalline polymers as a simple mixture of amorphous and crystalline regions, although they differ in the mathematical methods used for their calculations.^14,15^ These approaches satisfy the theoretical requirements, but their accuracy sometimes lacks experimental validation. In addition, these models require complex and time-consuming calculations and constants which are not known for most of the polymers. In many cases, the orientation and size distribution of the lamellar structure must be known for the aforementioned calculations, but these are difficult to determine. Accordingly, these methods cannot be applied routinely in everyday practice. Our previous research focused on the development of an empirical method to predict the tensile modulus of semicrystalline polymers based on parameters of the crystalline structure obtained from simple calorimetric measurements.^16–20^ Accordingly, the tensile modulus can be calculated as

1 $$E=E_{a}+\frac{E_{c}-E_{a}}{{1+\left({\left(\frac{1-X}{X}\right)}^{\alpha }+{\left(\frac{T_{m}^{0}-T_{av}}{T_{m}^{0}}\right)}^{\beta }\right)}^{\gamma }}$$

where *E* is the tensile modulus of a sample, *E*_a_ and *E*_c_ are the moduli of amorphous and crystalline phases, and *X* is the crystallinity. *T*_m_^0^ is the equilibrium melting temperature, *T*_av_ is the average melting point, and α, β, and γ are iterative empirical constants. The average melting temperature is proportional to the lamellar thickness of the polymer.^19^ Accordingly, eq 1 determines the tensile modulus based on two primary parameters, which are crystallinity and *T*_av_ (a lamellar thickness-related parameter).^21^ Crystallinity is calculated using the equilibrium enthalpy of fusion (*X* = Δ*H*_m_/Δ*H*_m_^0^), where Δ*H*_m_ and Δ*H*_m_^0^ are the experimental and equilibrium enthalpies of fusion, respectively. Δ*H*_m_^0^ can be taken from a reliable literature source. The advantage of this empirical model is its ease of use, because only equilibrium heat of fusion is required for the calculations. All other thermodynamic parameters can be obtained from a simple calorimetric melting curve.

The empirical approach proposed in our earlier studies was tested on polymers for which the mechanical properties are known to be governed predominantly by the crystalline structure. The basic principles are the same for polyamide-6, but its tensile modulus is also significantly influenced by the absorbed water. The present work therefore examines whether the previously developed model can also be applied to a hygroscopic polymer and how the effect of water content can be modeled quantitatively. To the best of our knowledge, similar easily applicable models for predicting the tensile modulus of PA6 as a function of water content are lacking in the literature, although such models could have great potential from both an industrial and scientific perspective. Accordingly, the novelty of our work lies in the physical interpretation of the empirical parameters in eq 1 and in the identification of their dependence on the water content of PA6.

## Experimental Section

### Materials and Sample Preparation

Akulon F223-D, an injection molding grade polyamide-6, was used for the study. The physical properties of the polymer are listed in Table 1.

**Table 1:** Physical Properties of Akulon F223-D-Type Polyamide-6

density (g/cm^3^)	tensile modulus (MPa)	melting point (°C)	water absorption (m/m %)	viscosity number (cm^3^/g) (ISO 307)	supplier
1130	3200–1000	220	10	132	Envalior Ltd.

The polymer was dried prior to processing at 80–100 °C for 4 h in an oven. Injection-molded test specimens (ISO 527-2) of polyamide 6 were made using a DEMAG IntElect 560/330-100-type electronic injection molding machine at 255–240–230–220 °C barrel and 80 °C mold temperatures, and 2000 bar injection and 450 bar holding pressures with 25 s cooling and holding time. The width and thickness of the produced specimens were 10 and 4 mm, respectively.

The produced test specimens were conditioned for about 27 days (650 h) in desiccators with different relative humidities (target values were 0–30–50–70–100%). Different relative humidity values were set using different saturated solutions. Due to the long conditioning, the equilibrium humidity values deviated from the target values; therefore, the actual values in equilibrium are shown in Table 2.

**Table 2:** Data of Relative Humidity in Desiccators

nominal relative humidity [%]	0%	30%	50%	70%	100%
actual relative humidity [%]	4%	36%	57%	73%	99.9%
solution	phosphorus pentoxide	magnesium chloride	magnesium nitrate	sodium chloride	distilled water

The specimens were placed in the desiccators, and the water content was measured frequently and calculated as follows

2 $$water\;content=\frac{m-m_{0}}{m}\times 100$$

where *m*_0_ is the weight of the test specimen before placing it in the desiccator and *m* is the measured mass of the test specimen in a given desiccator at the given time. The mass was measured in a Sartorius analytical balance with an accuracy of 10^–5^ g.

### Characterization Techniques

The melting characteristics were examined using a PerkinElmer Diamond DSC device. A high-purity N_2_ atmosphere (20 mL/min) was used as a purge gas. Samples between 3 and 5 mg were used during the measurements, and all samples were sealed hermetically in aluminum sample holder crucibles. All samples were heated from 30 to 290 °C at a heating rate of 10 °C/min. Although in conventional DSC measurements, the first heating run is usually used to eliminate thermal and mechanical prehistory, in our case, it is essential to use the melting curve recorded during the first heating because this melting peak refers to the crystalline structure studied during mechanical tests.

An Instron 5566-type standard tensile testing machine was used for the mechanical tests to determine the tensile modulus values of all the conditioned samples. The crosshead speed was 0.5 mm/min, which is a standard value for measuring the tensile modulus. The gauge length was set to the standard value of 115 mm. All evaluation and measurement procedures were performed according to the ISO 527-1:2019 standard.

PerkinElmer Diamond DMA was used to measure the glass transition temperature of the injection-molded specimens. The measurements were made using the standard ISO 527-1_2019 samples. A 40 mm long piece was cut from the center of the specimen and was inserted into the instrument. All measurements were done in single cantilever bending mode. The samples were cooled to −120 °C and held there for 10 min to stabilize the starting temperature. Subsequently, all samples were heated to 120 °C at a heating rate of 2 °C/min. The periodic frequency was 1 Hz, and the instrument was purged by nitrogen gas at a rate of 80 mL/min. We have to note that the water content may change a little during the measurement, since the relative humidity could not be controlled within the DMTA Instruments.

## Results and Discussion

The water uptake of the injection-molded specimens is presented in Figure 1. The data presented here demonstrate clearly that water content increases remarkably, especially at high relative humidity, and reaches a saturation value within 25 days if the relative humidity is lower than 70%. Accordingly, 25 days were long enough to reach the dynamic equilibrium in the case of 36 and 57% of relative humidity, respectively.

**Figure 1:**
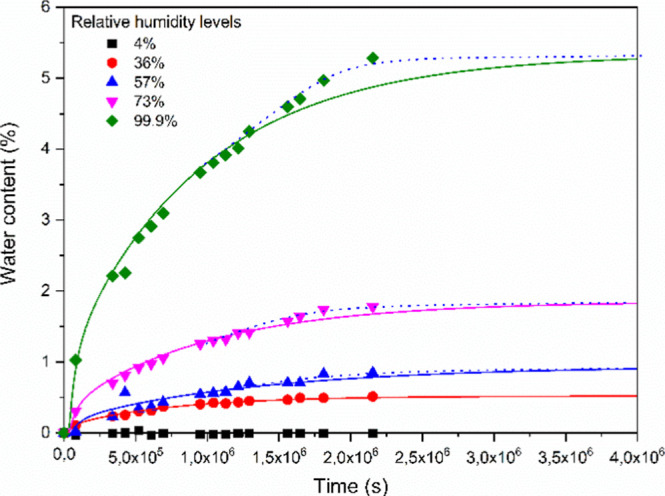
Water content of polyamide-6 samples in different relative humidities. The solid lines show fits of the experimental data using a Fickian-type diffusion model, while the dashed lines are free-hand drawn curves indicating deviations from Fickian behavior.

Water uptake of polyamides can be modeled by fitting Fick’s law to the experimental curves. Fick’s second law of diffusion (eq 3) describes the accumulation of water in a sample as a function of time with a diffusion coefficient of *D*. In the case of diffusion through a given surface, the general Fick equation can be written in a simpler form as eq 4, and the diffusion coefficient can be determined from the slope at a given time.^22^

3 $$\frac{\partial c}{\partial t}=\frac{\partial }{\partial x}\left(D\frac{\partial c}{\partial x}\right)$$

4 $$M_{t}=2\times A_{tot}\times \sqrt{\frac{Dt}{\pi }}$$

*M*_*t*_ is the mass increase of the sample at time *t*, and *t* is the time range in seconds. *A*_tot_ is the total surface area of the specimen. However, the simplified expression of diffusion (eq 4) does not consider two important issues. First, saturation, namely, the equilibrium between adsorption and desorption, is not included in eq 4. Moreover, in polyamides, the adsorption of water influences intermolecular interactions in the polymer. Accordingly, the glass transition temperature (*T*_g_) of the polymer also decreases during conditioning. PA6 is an example of those polymers where *T*_g_ decreases steeply with water uptake, and it can decrease even below room temperature, where usually the conditioning takes place. Consequently, the physical state of the polymer is changing during conditioning, and the diffusion coefficient will also change if the amorphous phase enters the rubbery state. In this case, Fick’s law, which uses the diffusion coefficient as a constant quantity, cannot be used reliably, and the experimentally recorded water uptake curves may deviate from a Fickian behavior (see the free-hand drawn dashed curves in Figure 1). The non-Fickian water absorption was modeled by Arhant et al.,^23^ but they also revealed that the saturation level modeled by Fickian or non-Fickian equations was the same at 25 °C. Taking these issues into account, we used a Fickian equation, which considers desorption as well, to describe the saturation level accurately because our goal was to determine the tensile modulus in saturated samples (see eq 5).^24^

5 $$M_{t}=M_{\infty }\left(1-\frac{8}{\pi^{2}}\left(e^{-at}+\frac{e^{-9at}}{9}+\frac{e^{-25at}}{25}\right)\right)$$

The variables of *M*_*t*_ and *M*_∞_ are the water uptake in percentage at a given time or at saturation, respectively. The constant *a* characterizes the overall rate of water adsorption, and *t* is the time. All predicted *M*_∞_ values and the experimentally recorded water contents are collected in Table 3, and it can be established that the experimental values are very close to the final saturation values. Accordingly, it can be stated that the samples reached saturation within 25 days of storage in all conditions.

**Table 3:** Water Content of Polyamide-6 Samples Measured at 25 Days or Predicted for Equilibrium

relative humidity [%]	water content [%] at 25 days	*M*_∞_ [%] predicted for equilibrium
4	0^a^	-
36	0.51^a^	0.52
57	0.85	0.94
73	1.78	1.85
99.9	5.29	5.34

a Equilibrium values estimated at relative humidity levels lower than 57%.

It should be noted that in the case of 57, 73, and 99.9% relative humidities, the non-Fickian behavior indicates that the adsorption of water accelerates, so we may suppose that the glass transition of the samples decreased remarkably due to the increasing water content. Therefore, the glass transition as a function of the water content is presented in Figure 2.

**Figure 2:**
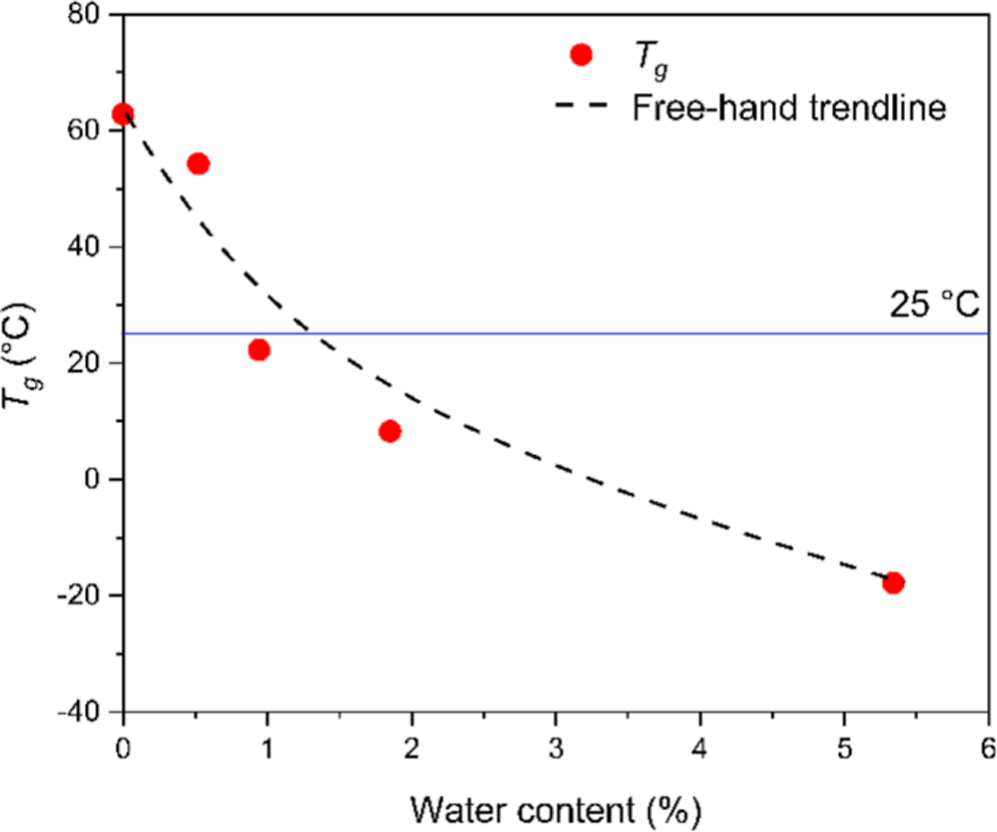
Change in *T*_g_ values as a function of water content.

The *T*_g_ values were determined by the DMTA method and taken as the peak temperatures of the tan δ curves. The results presented in Figure 2 clearly indicate that the drop of *T*_g_ is reasonably large, and in the case of 57% relative humidity and above, the glass transition temperature decreased below room temperature. Accordingly, the amorphous phase is in a rubbery state, where molecular mobility and transport processes are accelerated, which is accompanied by faster water uptake and deviation from Fickian behavior. The deviation becomes monotonously larger with the decrease of *T*_g_. We have to call attention to the fact that the accelerated water uptake does not influence the equilibrium water content, as was also shown earlier.^23^

Melting characteristics as well as crystallinity data evaluated from the DSC melting curves are collected in Table 4. It can be clearly seen that the crystallinity of the samples is around 30% and the average melting temperature is around 216–217 °C. The results indicate clearly the fact that the water content does not influence significantly the melting characteristics of the sample. Although it is known that the water content may influence the crystalline structure if it is present during the processing of the polymer, if water is absorbed by diffusion after the crystalline phase has formed, it is taken up by the amorphous phase and does not influence the crystalline phase. Accordingly, we may assume that mechanical properties, especially the modulus, are affected by the water content because the water incorporates in the amorphous phase and replaces the intermolecular hydrogen bonds by molecule–water H-bonds; therefore, the intermolecular cohesion forces decrease significantly; consequently, the modulus also decreases proportionally.^8,9^ The crystallinity values were calculated using the equilibrium enthalpy of fusion value of polyamide-6 at 229.8 J/g.^25^

**Table 4:** Melting Characteristics and Crystallinity Data of Polyamide-6 Samples Conditioned at Different Humidities

relative humidity [%]	*T*_av_ (°C)	st. dev. of *T*_av_ (°C)	*X* (-)	dev(*X*)
4	217.2	0.7	0.31	0.003
36	216.9	0.2	0.30	0.005
57	217.2	0.6	0.30	0.004
73	216.8	0.8	0.31	0.011
99.9	216.9	0.6	0.28	0.001

The tensile modulus values as a function of the water content are presented in Figure 3. The results indicate that a remarkable decrease can be observed in the tensile modulus if water content increases in polyamide-6.^7^ Although this phenomenon is well-known, its qualitative prediction is much less described in the literature. It seems that the presence of water in the amorphous phase of polyamide-6 has a crucial effect from the point of view of the modulus and other mechanical properties as well.

**Figure 3:**
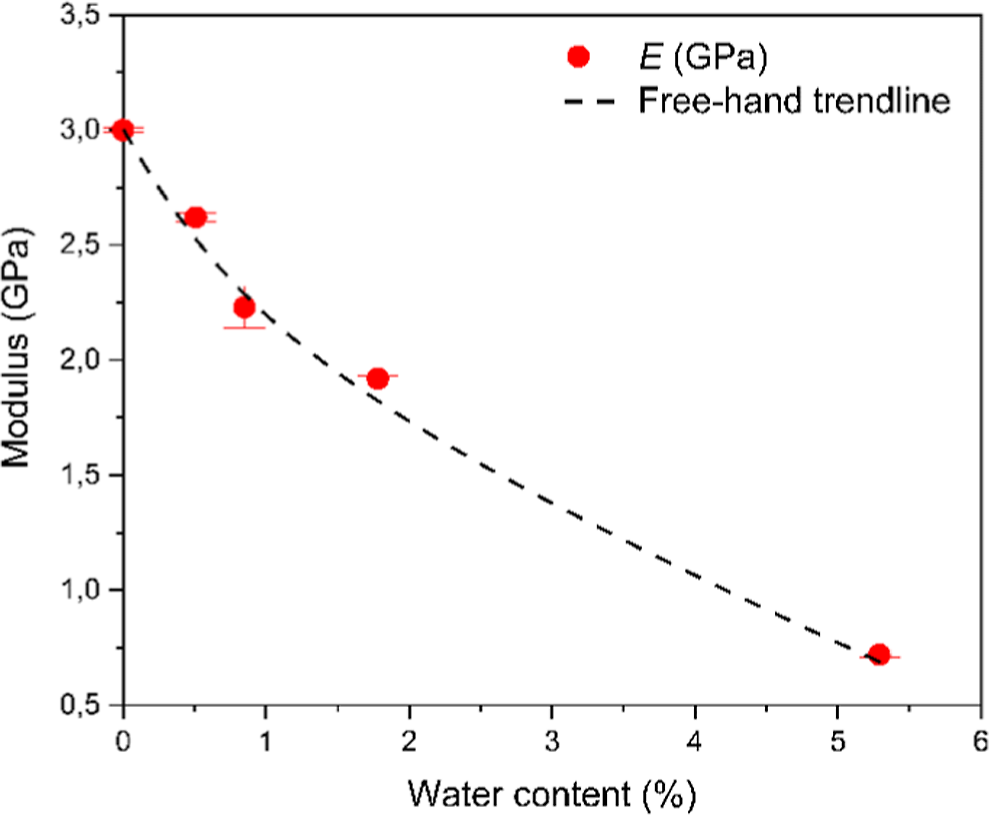
Change in the tensile modulus as a function of water content.

We used data from our earlier studies^19^ as well as some unpublished works and fitted eq 1 to these data points using the multiple nonlinear regression method using the Origin Pro Software package. The results of the fitting procedure are presented in Figure 4. The data taken from our earlier unpublished studies are given in the Supporting Information (Tables S1–S3).

**Figure 4:**
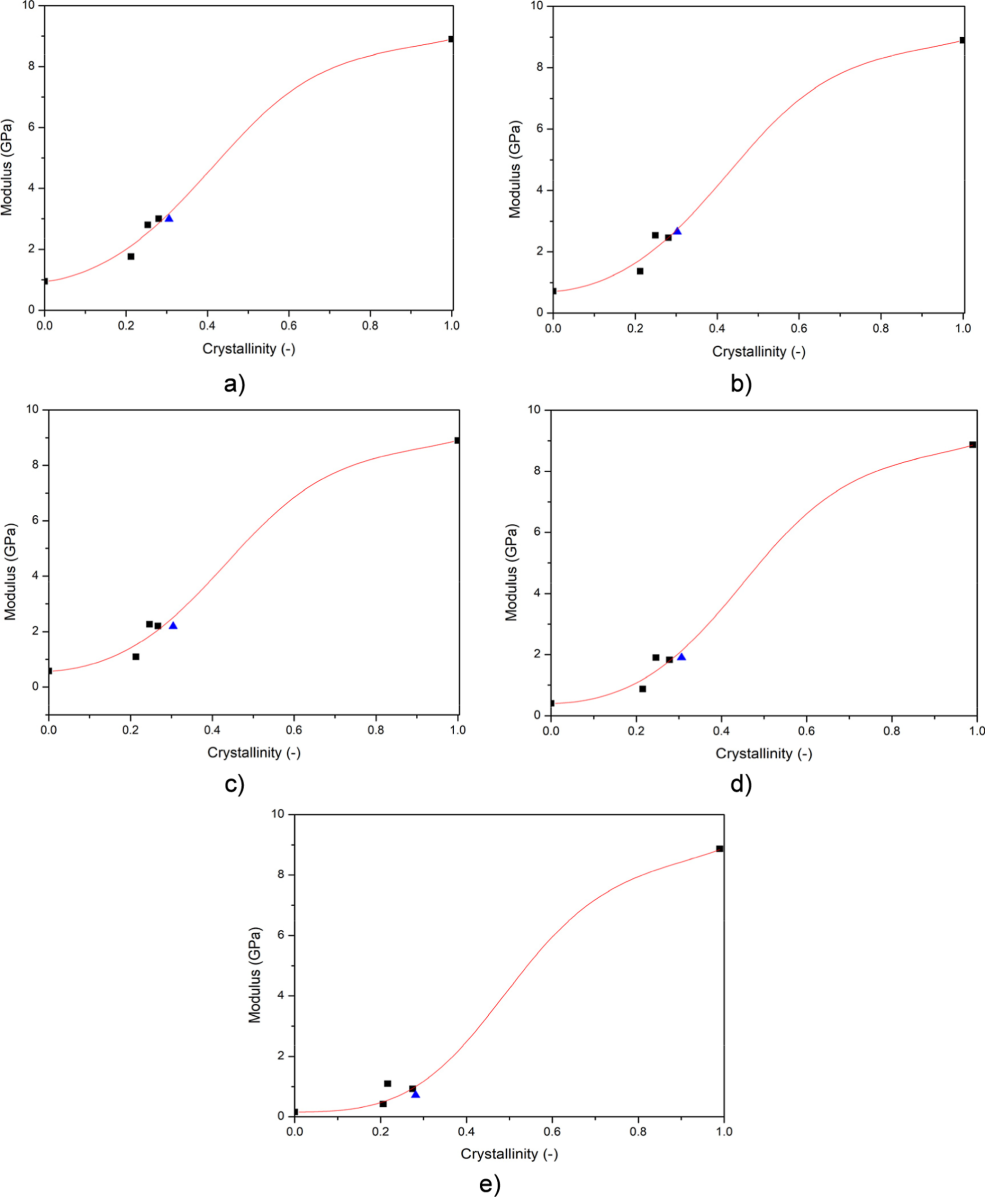
Fitting results of eq 1 to experimental points obtained on specimens conditioned at (a) 0%, (b) 36%, (c) 57%, (d) 73%, and (e) 99.9% relative humidities. Previous studies (■); this study (▲) (*R* ^2^ is always above 0.99)

The data obtained in this study (blue triangles in Figure 4) fit well with earlier results, indicating that our model is usable and reliable for different polyamide-6 grades with the same parameter set. Unfortunately, the crystallinity data presented in these figures hint that polyamide-6 has relatively low crystallinity, which cannot be changed over a wide range. This, unfortunately, limits the reliable application of our empirical approach to a limited range of crystallization that is close to the experimental data. The reliability can be enhanced, of course, if a wider crystallinity range is covered by experimental points during the fitting procedure. However, despite the limitation of this approach in the case of polyamide-6, our prediction model can be used reliably in the range of practical crystallinity of commercial polyamide grades, since their crystallinity is within 20–30%, which overlaps with the crystallinity of our earlier experimental points, and in this region, the empirical prediction is reliable. We have to highlight also that the modulus value of totally amorphous polyamide-6 is also not a constant because it strongly depends on water content. Accordingly, *E*_a_ values were also estimated as variables because totally amorphous polyamide-6 cannot be produced either. The modulus of the crystalline phase was taken from our earlier publication, and it is 8.9 GPa.^19^ It was calculated from the propagation of the sound waves in polyamide-6 according to the model proposed by van Krevelen et al.^26^ All of the estimated values obtained from the fitting procedure are presented in Table 5. *T*_m_^0^ was taken from the book of Wunderlich.^25^

**Table 5:** Values and Iterative Constants Used and Obtained during Fitting of Equation 1 to Experimental Data Presented in Figure 4

humidity [%]	*E*_c_ [GPa]	*E*_a_ [GPa]^a^	*T*_m_^0^ [°C]_PA6_	α^a^	β^a^	γ^a^
4	8.9	0.95	260	2.4	0.9	0.6
36	8.9	0.72	260	2.4	0.9	0.65
57	8.9	0.57	260	2.4	0.9	0.68
73	8.9	0.4	260	2.4	0.9	0.75
99.9	8.9	0.16	260	2.4	0.9	0.99

a Estimated by iteration.

We have to note that only *E*_a_, α, β, and γ were estimated by iteration. In addition, we decided to fix *E*_c_, α, and β parameters for fitting to all different water contents because these constants are connected to the crystalline phase, which does not change under different relative humidity conditions (see all data in Table 4). We also suppose that the iterative constants α, β, and γ, although they are numbers without deep physical meaning, represent weighting factors of crystallinity, lamellar structure, and intermolecular interactions, respectively. Since crystallinity and lamellar thickness do not change, α and β were kept constant, whereas γ was defined as a function of water content. Accordingly, two parameters are strongly dependent on water content, namely, the *E*_a_ and γ parameters. If this assumption is true, we have to check the correlation between water content and the γ parameter or *E*_a_. These correlations are presented in Figure 5.

**Figure 5:**
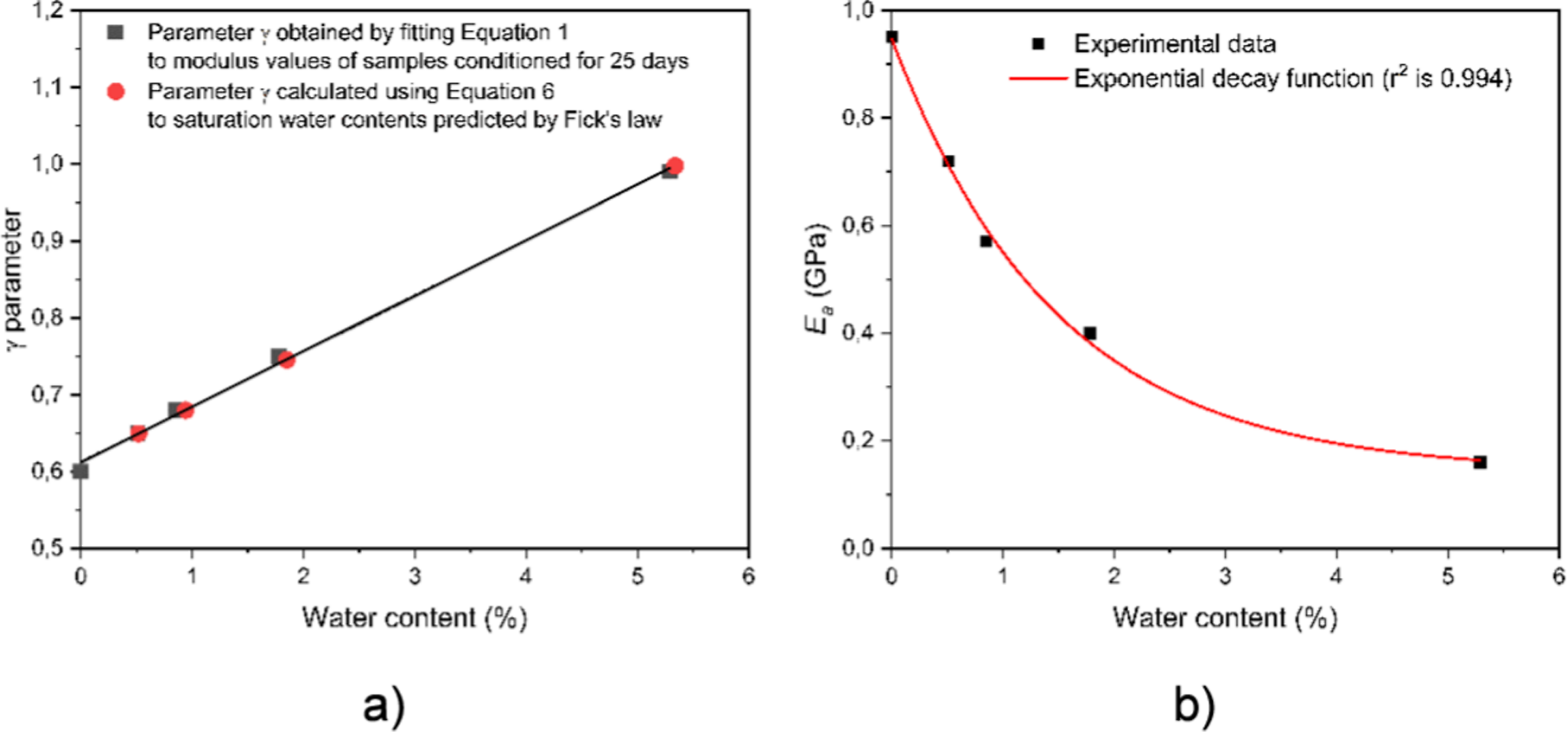
The correlation between water content and (a) γ parameter and (b) iterated *E*_a_ values.

As can be seen in Figure 5, a strong linear correlation exists between the estimated γ parameter and the water content based on the experimentally recoded data points. In addition, the modulus of the amorphous phase depends exponentially on water content. This correlation supports our assumption that the γ parameter weights the intermolecular interactions in the polymer. If the intermolecular interactions are modified by water or any other additive, this parameter also changes accordingly. The weaker the interaction between the molecules, the greater the increase in the γ parameter. A linear equation was fitted to the water content data points, and an exponential equation was fitted to the *E*_a_ data points (eqs 6 and 7). Using the equations obtained from Figure 5, the γ parameter and *E*_a_ can be calculated as follows

6 $$\gamma =0.612+0.0722W_{c}$$

7 $$E_{a}=E_{a}^{0}+0.8{1e}^{{-W}_{c}/1.48}$$

where *W*_c_ is the water content in percentage and *E*_a_^0^ is the theoretical (extrapolated) value for the modulus at infinite water content, which is equal to 0.14. We assumed that the linear correlation also holds over a wider range of water contents. Therefore, we used eq 6 to calculate the γ parameter for water content predicted by Fick’s law for saturation levels as well, although these values are always close to the experimental ones. These points are presented as red dots in Figure 5a. Since water is absorbed mainly in the amorphous phase, the change of the mechanical properties, especially the decrease of modulus, is also not negligible. Figure 5b presents the drop in the modulus value as a function of equilibrium water content. As can be seen, the lowest extrapolated modulus value is 0.14 GPa.

Accordingly, if the water content, crystallinity, and average melting point of polyamide-6 are known, the modulus can be calculated easily. In addition, the tensile modulus can be modeled in a wide range of water content in polyamide-6 specimens. The modulus values were calculated for the saturation water content, which were predicted by Fick’s law, and these points are plotted together with the experimentally recorded tensile modulus values as a function of water content (Figure 6). A generally good agreement is observed between predicted and measured modulus values over a wide range of water contents, although one data point shows a noticeable deviation. Accordingly, we can establish that the modeling of saturation water content was reliable using eq 5. The simulated tensile stiffness data are also presented in Figure 6 as a continuous blue line, and it can be seen that the simulation follows the experimental data reasonably well. In fact, most of the points fall within 10% of the deviation band, indicated by the dotted lines, which can be considered good agreement for the prediction of mechanical properties. Accordingly, eq 1 and our simple empirical model are applicable for modeling the properties of polyamide-6 as a function of water content. We may speculate why the experimental point at high water content does not agree well with the prediction, but we have to note here that measuring the mechanical properties of saturated PA6 is challenging, since the properties of the sample can also change during the test due to water desorption. In addition, the simulation was performed using fixed values of crystallinity and *T*_av_, selected as 0.305 and 217 °C, respectively. However, this crystallinity is higher than that of the sample conditioned at 100% relative humidity (see Table 4). The value of *X* = 0.3 is closer to the crystallinity of all the other samples; therefore, the simulation shows good agreement with those samples.

**Figure 6:**
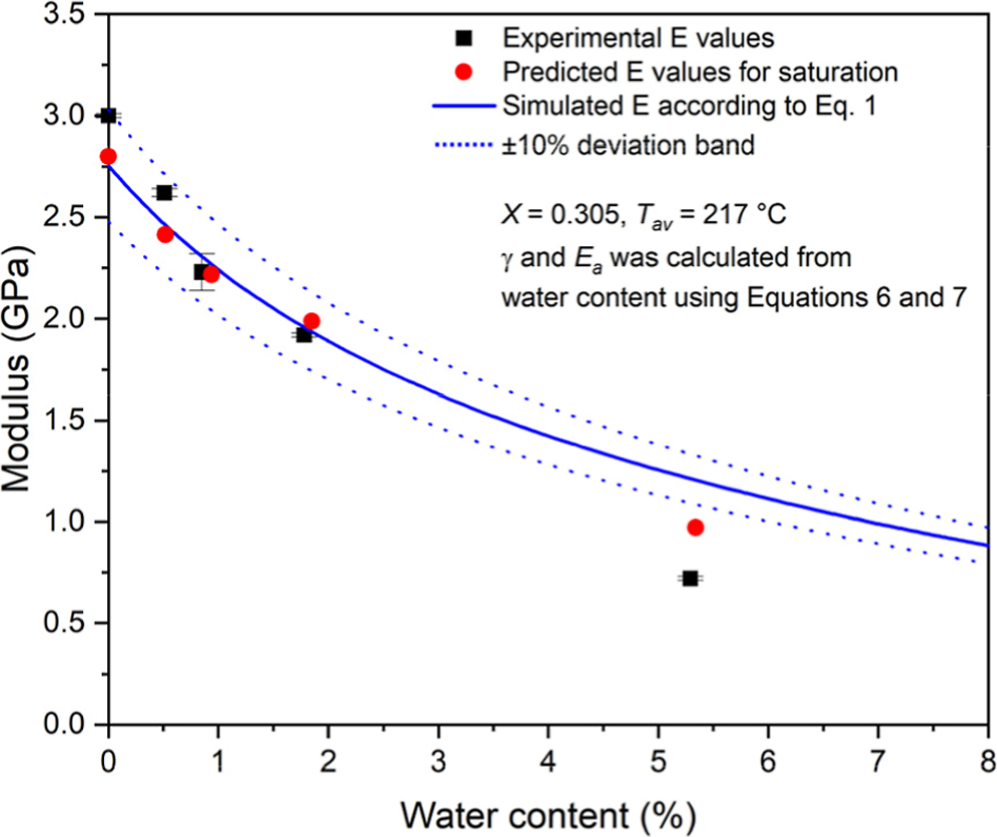
Decrease of modulus values in a wide water content range and simulated modulus values using eq 1 (α and β were taken from Table 4).

As a final validation, calculated and experimentally measured modulus values are plotted against each other, and the results are presented in Figure 7. The good correlation between the results is convincing and demonstrates clearly that our empirical approach can be used even if the fitting was made on data sets in a narrow range of crystallinity. We have to note here as well that small deviations from the model are observed even at totally wet or dry conditions, most probably because of the dynamic change of the properties of the specimens even during the testing procedures. However, we have to highlight here that such fitting is reliable only close to the crystallinity range of samples used for the experiments. If experimental data are available over a wider crystallinity range, like in the case of polypropylene, poly­(lactic acid), poly­(ethylene-terephthalate), and polyethylene,^16–18^ this empirical approach can be used for the prediction of a material’s limitations over a wider range of crystallinity.

**Figure 7:**
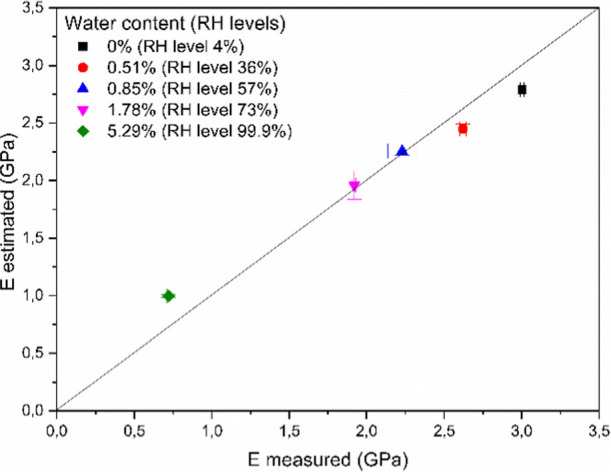
Measured and predicted modulus values of samples conditioned at different relative humidity levels.

## Conclusion

This study aimed to model the tensile modulus of polyamide-6 as a function of its water content. In order to study the change of stiffness as a function of absorbed water, samples were conditioned under different relative humidity values and their tensile properties were measured after 25 days of conditioning. Based on the Fickian diffusion equation, it was proven that the samples reached the saturation water content during the conditioning. Our empirical approach, developed earlier for the prediction and modeling of tensile modulus, was used to describe the dependence of stiffness on water content quantitatively. It was found that the water content in polyamide-6 changes continuously during conditioning, and reaching saturation can take longer, especially at higher relative humidity levels. The results proved clearly that the adsorption of water obeys the second Fick’s law below a 57% relative humidity, but if the relative humidity is above 50%, the adsorption follows a non-Fickian trend. The results of the calorimetric study revealed that the crystalline structure does not change remarkably during conditioning, and water is absorbed predominantly in the amorphous phase. Accordingly, the presence of water significantly changes the intermolecular hydrogen bonding in the amorphous phase, and consequently, the tensile modulus also changes. We found that the iterative constant of γ depends linearly on water content in a wide range and can be considered as the weighing factor of intermolecular interactions. Using these correlations, the tensile modulus is modeled successfully in a wide range of water content even if water content was predicted using Fick’s law. Accordingly, this method makes it possible to model the tensile modulus of any polyamide-6 grade quantitatively in a wide range of water content based on a simple melting curve.

## Supplementary Material



## Notes

Main properties and earlier unpublished data for Polyamide 6 (Promyde B30 P2 and Promyde B15 P) (DOCX)

## References

[1] Gilbert, M. Chapter 18-Aliphatic Polyamides. In Brydson’s Plastics Materials, 8th ed.; Gilbert, M. , Ed.; Butterworth-Heinemann, 2017; pp 487–511.

[2] Matthies, P. ; Seydl, W. F. History and Development of Nylon 6, High Performance Polymers; Their Origin and Development: Dordrecht, 1986; pp 39–53

[3] Carothers W. H., Hill J. W. (1932). Studies of polymerization and ring
formation; xiii.
Polyamides and mixed polyester-polyamides. J.
Am. Chem. Soc..

[4] Galanty, P. G. , Jacob, K. I. Nylon 6. In Polymer Data Handbook: 2nd ed.; Mark, J. E. , Ed., Oxford University Press, 2009.

[5] Holmes D. R., Bunn C. W., Smith D. J. (1955). The crystal structure of polycaproamide:
Nylon 6. J. Polym. Sci..

[6] Huang H.-X., Wang B., Zhou W.-W. (2012). Polymorphism
in polyamide 6 and polyamide
6/clay nanocomposites molded via water-assisted injection molding. Composites, Part B.

[7] Miri V., Persyn O., Lefebvre J. M., Seguela R. (2009). Effect of water absorption
on the plastic deformation behavior of nylon 6. Eur. Polym. J..

[8] Shinzawa H., Mizukado J. (2020). Water absorption by
polyamide (PA) 6 studied with two-trace
two-dimensional (2T2D) near-infrared (NIR) correlation spectroscopy. J. Mol. Struct..

[9] Silva L., Tognana S., Salgueiro W. (2013). Study of the
water absorption and
its influence on the Young’s modulus in a commercial polyamide. Polym. Test..

[10] Halpin J. C., Kardos J. L. (1972). Moduli of Crystalline
Polymers Employing Composite
Theory. J. Appl. Phys..

[11] Fukahori Y., Hon A. A., Jha V., Busfield J. J. C. (2013). MODIFIED GUTH–GOLD
EQUATION FOR CARBON BLACK–FILLED RUBBERS. Rubber Chem. Technol..

[12] Boyd, R. H. Prediction of Polymer Crystal-Structures and Properties. Atomistic Modeling of Physical Properties; Springer, 1994; Vol. 116, pp 1–25.

[13] Boyd R. H. (1983). The Mechanical
Moduli of Lamellar Semicrystalline Polymers. J. Polym. Sci., Part B: Polym. Phys..

[14] Curtis P. T., Bader M. G., Bailey J. E. (1978). The stiffness and
strength of a polyamide
thermoplastic reinforced with glass and carbon fibres. J. Mater. Sci..

[15] Mori T., Tanaka K. (1973). Average stress in matrix
and average elastic energy
of materials with misfitting inclusions. Acta
Metallurgica.

[16] Zarbali A., Djaffar I., Menyhárd A. (2024). Prediction
of tensile modulus based
on parameters of crystalline structure in polyethylene terephthalate
with cold crystallization ability. Heliyon.

[17] Zarbali A., Pinke B., Menyhárd A. (2023). Robustness
study of a tensile modulus
prediction model for semicrystalline polymers. Period. Polytech., Chem. Eng..

[18] Molnár J., Hertner-Horváth A., Menyhárd A. (2021). Prediction
of tensile modulus from calorimetric melting curves of polylactic
acid with pronounced cold crystallization ability. Polym. Test..

[19] Molnár J., Jelinek A., Maloveczky A., Móczó J., Menyhárd A. (2018). Prediction of Tensile Modulus of
Semicrystalline Polymers
From a Single Melting Curve Recorded by Calorimetry. J. Therm. Anal. Calorim..

[20] Menyhárd A., Suba P., László Z., Fekete H. M., Mester A. ´. O., Horváth Z., Vörös G., Varga J., Móczó J. (2015). Direct Correlation
Between Modulus
and the Crystalline Structure in Isotactic Polypropylene Express. Polym. Lett..

[21] Pukánszky B., Mudra I., Staniek P. (1997). Relation of
Crystalline Structure
and Mechanical Properties of Nucleated Polypropylene. J. Vinyl Addit. Technol..

[22] Vlasveld D. P. N., Groenewold J., Bersee H. E. N., Picken S. J. (2005). Moisture absorption
in polyamide-6 silicate nanocomposites and its influence on the mechanical
properties. Polymer.

[23] Arhant M., Le Gac P.-Y., Le Gall M., Burtin C., Briançon C., Davies P. (2016). Modelling the non Fickian water absorption
in polyamide
6. Polym. Degrad. Stab..

[24] Müller P., Renner K., Móczó J., Fekete E., Pukánszky B. (2014). Thermoplastic
starch/wood composites: Interfacial interactions and functional properties. Carbohydr. Polym..

[25] Wunderlich, B. Thermal Analysis of Polymeric Materials; Springer: Berlin, 2005.

[26] van Krevelen, D. V. ; Nijenhuis, K. T. Properties of Polymers; Elsevier: Amsterdam, 2009.

