# Supplementary material for: Empirical Modeling of the Effect of Water Content on the Tensile Modulus of Polyamide‑6
Source: ACS Omega. 2026 May 20;11(21):31292–9. doi: 10.1021/acsomega.6c01154 (PMC13234804; doi:10.1021/acsomega.6c01154)
Supplement: Supplementary file 1 [file ao6c01154_si_001.docx]

**Empirical modeling of the effect of water content on the tensile modulus of polyamide-6**

**Supplementary data**

Ali Zarbali^^[[1]](#footnote-1)^*^, Alina Tilekkabylova^1^, Alfréd Menyhárd‬^1^

^1^Laboratory of Plastics and Rubber Technology, Department of Physical Chemistry and Materials Science at Budapest University of Technology and Economics, Műegyetem rkp. 3. H/1, Budapest 1111, Hungary

This supplementary file presents unpublished modulus data obtained during thesis work conducted in our laboratory. Although these measurements were not part of the recent study, they are included here to expand the dataset and allow a more comprehensive evaluation of the predictive model.

**Table S1**. Main properties of Polyamide 6, **Promyde® B30 P2** (NUREL, S.A.)

| Property | Unit | Value | Standard |
| --- | --- | --- | --- |
| Melt volume-flow rate | cm^3^/10min | 70 | ISO1133 |
| Density | g/cm^3^ | 1.08 | ISO1183 |
| Tensile modulus | GPa | 2.2 | ISO527-1/-2 |
| Melting point | °C | 222 | ISO3146 |
| Water absorption | m/m % | 7.6 | ISO 62 |

**Table S2**. Main properties of Polyamide-6, **Promyde® B15 P** (NUREL, S.A.)

| Property | Unit | Value | Standard |
| --- | --- | --- | --- |
| Melt volume-flow rate | cm^3^/10min | 260 | ISO1133 |
| Density | g/cm^3^ | 1.13 | ISO1183 |
| Tensile modulus | GPa | 3.3 | ISO527-1/-2 |
| Melting point | °C | 222 | ISO3146 |
| Water absorption | m/m % | 9.5 | ISO 62 |

**Table S3.** Experimental modulus values for different PA6 grades conditioned at varying water contents, obtained from previous work

| Actual relative humidity during conditioning (%) | **Promyde® B15 P** | **Promyde® B30 P2** |
| --- | --- | --- |
| 4 | 1.76 ± 0.02 GPa | 3.00 ± 0.05 GPa |
| 36 | 1.37 ± 0.04 GPa | 2.46 ± 0.02 GPa |
| 57 | 1.09 ±0.01 GPa | 2.21 ± 0.02 GPa |
| 73 | 0.87 ± 0.02 GPa | 1.83 ± 0.04 GPa |
| 99.9 | 0.42 ±0.01 GPa | 0.93 ± 0.04GPa |

1. *Corresponding author

   Email address: ali.zarbali@edu.bme.hu [↑](#footnote-ref-1)
